# Coupled CFD-DEM simulation of progressive failure of tunnel face in sand

**DOI:** 10.1371/journal.pone.0319184

**Published:** 2025-03-11

**Authors:** Zuyan Wang, Chuang Zhou, Yongkang Zhang, Weiyi Li, Yongjun Qin, Jiangu Qian

**Affiliations:** 1 College of Civil Engineering and Architecture, Xinjiang University, Urumqi, China; 2 Department of Geotechnical Engineering, Tongji University, Shanghai, China; 3 Department of Science and Technology Quality, CCFEB Civil Engineering Co., Ltd., Changsha, Hunan, China; NED University of Engineering and Technology, PAKISTAN

## Abstract

Many studies have investigated tunnel face failure using the discrete element method (DEM). However, DEM simulations that incorporate water-soil interactions in tunnel face failure are still limited. In this study, the coupled CFD-DEM method is employed to simulate the progressive failure of the shield tunnel face in both saturated and dry sand. The dynamic mesh method is applied in the CFD component to accurately simulate the changes in the CFD domain due to the movement of the tunnel face. The excavation face is moved forward or backward at a uniform rate to simulate passive and active failure, respectively. The analysis focuses on progressive failure, ground surface displacement, tunnel face support forces, force chain distribution, and vertical stress distribution. Our results at both macro- and micro-scales reveal significant effects of soil-water interactions.

## 1. Introduction

The shield tunneling method is commonly used in urban underground tunneling projects. Inadequate or excessive support forces on the tunnel face in sand can lead to tunnel destabilization, resulting in various engineering issues [1–4], such as excessive surface settlement or cracking of buildings near the tunnel.

A series of studies on tunnel face failure have been conducted, primarily focusing on three aspects: theoretical solutions, experimental studies, and numerical simulations. In terms of analytical solutions, Horn [5] proposed the classical ‘wedge’ model for assessing tunnel face stability based on the limit equilibrium principle. Anagnostou and Kovári [6] investigated the mechanics of face failure when using bentonite slurry retaining walls based on Horn’s theory [5]. Leca and Dormieux [7], as well as Mollon et al. [8], applied the limit analysis method to provide an upper limit solution for the ultimate support force of a 3D tunnel excavation. Zou et al. [9] developed a new model for assessing tunnel face stability, incorporating the soil arch effect.

A significant number of researchers have studied tunnel face failure using experimental methods. For example, Chen et al. [10] conducted a series of model tests with varying C/D ratios to investigate the failure behavior of shield tunnels in sandy soil. Lü et al. [11] performed physical model tests on tunnel face stability in anisotropic granular media, identifying the failure modes of tunnel faces. Ohta et al. [12] experimentally demonstrated that the collapse morphology of tunnel faces in sandy strata is influenced by surface characteristics and groundwater conditions. Oblozinsky and Kuwano [13] developed a half-tunnel model, using photographs of soil layers at pre-buried marker points to reveal the mechanism of progressive destruction. Chambon and Corte [14] conducted centrifugal model tests in sandy soils, investigating the influence of soil density, tunnel location, and tunnel excavation face diameter on the ultimate support force. Idinger et al. [15] explored the effect of tunnel burial depth on the failure pattern in loose sandy soil using digital photography.

Numerical simulation methods are primarily classified into continuous medium methods (e.g., finite element method, finite difference method) and discrete element methods. In the context of continuous medium methods, Ibrahim et al. [16] applied the finite element method to simulate tunnel excavation face instability in dry, multilayered cohesionless soil, verifying a new mechanism of face failure. However, in the region near the tunnel face, where progressive failure occurs, large soil deformation or even soil collapse is common, which poses significant challenges for traditional continuous medium simulation methods. As a result, some researchers [17–20] have turned to discrete element methods to simulate the large deformation and soil transport, which provide insights into the microscopic mechanisms underlying macroscopic tunnel excavation face instability.

Nevertheless, previous DEM studies have generally been limited by computing power, which typically restricts particle count to no more than 100,000, making it difficult to accurately capture the damage patterns on the tunnel excavation surface. Furthermore, most discrete element numerical simulations do not account for water-soil interaction and typically simulate tunneling in dry sand. Zeng et al. [21] simulated saturated sandy soils by subtracting the buoyancy component from gravity. However, this simplification is overly simplistic, as it only considers the buoyancy forces acting on soil particles. In reality, water-soil interaction is a complex, multidisciplinary process governed by the principles of soil mechanics and hydraulics. Therefore, combining computational fluid dynamics (CFD) with the discrete element method (DEM) provides a more appropriate approach for modeling these interactions.

This paper simulates the progressive failure of a shield tunnel face in two types of soils: saturated sand and dry sand, using GPU-accelerated computing technology and an unresolved CFD-DEM approach with a particle count of 500,000. By comparing and analyzing the soil movement, surface displacement, and development of face support force, the influence of water-soil interaction on the progressive failure of the tunnel face is investigated. Additionally, the force chain and stress distribution in the progressive damage state of the tunnel are analyzed, revealing the fine-scale mechanisms in both saturated sand and dry sand cases.

## 2. Methodology of coupled CFD-DEM

### 2.1. Governing equations

The simulations presented in this paper are enabled by the integration of CFD and DEM. The coupling scheme was developed by Kloss et al. [22] and has been widely adopted in various soil-water interaction studies [23–27]. A brief overview of the numerical framework is provided below.

The motion of the solid particles is governed by Newton’s laws, while the velocity and pressure of the fluid are governed by the volume-averaged Navier-Stokes equations:


$$m_{i}\frac{dv_{i}}{dt}=\sum_{j}F_{ij}^{c}+\sum_{j}F_{ij}^{a}+F_{f\to p}+m_{i}g$$ (1)

$$I_{i}\frac{d\omega_{i}}{dt}=\sum_{j}M_{ij}^{c}+M_{f\to p}$$ (2)

$$\frac{\partial }{\partial t}\left(n\rho_{f}\right)+\nabla \cdot \left(n\rho_{f}u\right)=0$$ (3)

$$\frac{\partial }{\partial t}\left(n\rho_{f}u\right)+\nabla \cdot \left(n\rho_{f}uu\right)=-n\nabla p+\nabla \cdot (nT_{f})+n\rho_{f}g+F_{p\to f}$$ (4)

where $m_{i}$ is the particle's mass, $v_{i}$ and $\omega_{i}$ represents the translational and angular velocity, ***g*** is the gravitational acceleration vector. $F_{ij}^{c}$, $F_{ij}^{a}$ and $M_{ij}^{c}$ are the contact force, adhesive force and contact torque acting on particle *i* by particle *j* or the wall(s), respectively. $I_{i}$ is its moment of inertia tensor. Due to the fluid interaction, two additional terms appear when comparing with a pure DEM simulation: $F_{f\to p}$ is the additional force accounting for the interaction with the fluid phase and $M_{f\to p}$ is the additional torque due to the fluid phase velocity gradient. *n* is the porosity of the medium, $\rho_{f}$ is the density of fluid, *u* is the fluid phase velocity vector, *p* is the fluid pressure, $T_{f}$ is the stress tensor of the fluid phase. $F_{p\to f}$ indicates the forces generated by the particles on the fluid, calculated according to the expression:


$$F_{p\to f}=-\sum_{i=1}^{N}F_{f\to p}/\Delta V$$ (5)

where ΔV is the volume of a CFD cell, *N* is the particle number inside the cell [28,29]. More details of the calculation principles can be found in these studies [19,23,24,26,30].

### 2.2. Particle-fluid interaction forces

The fluid interaction force, $F_{f\to p}$, is commonly split into two terms: the drag force, $F_{D}$, and a second term composed by the remaining (non-drag) forces, $F_{\nabla p}$, in the following way:


$$F_{f\to p}=F_{D}+F_{\nabla p}$$ (6)

For cases where the mass ratio of the two phases is sufficiently small (for example, $m_{f}\ll m_{p}$), only drag and pressure gradient forces need to be considered. The pressure gradient force, $F_{\nabla p}$, is calculated according to the expression:


$$F_{\nabla p}=-V_{p}\nabla_{p}$$ (7)

where $V_{p}$ represents the volume occupied by solid particles, and $\nabla_{p}$ represents the average local pressure gradient.

Various drag force ($F_{D}$) models have been proposed in previous studies [31–35]. In the context of this study, the drag force for dense particle flows needs to be corrected based on the fluid volume fraction, in accordance with the single-particle law [36–38]. However, to date, no consensus has been reached among scholars regarding the drag force model for spherical particles in dense particle flows, although several established equations can still be applied in CFD-DEM simulations [30,39–41]. This paper adopts the drag force model proposed by Huilin and Gidaspow [42], which builds upon the work of Gidaspow et al. [43] and offers flexibility in switching between the Wen & Yu model and the Ergun model [44–46]. The model is expressed as [47]:


$$F_{D}=\frac{1}{2}C_{D}\rho_{f}A^{\prime }|u-v_{p}|(u-v_{p})$$ (8)

where $u-v_{p}$ represents the relative velocity between the particles and the fluid, $A^{\prime }$ represents the projected area of the particles in the direction of flow, and $C_{D}$ is the modified drag coefficient, calculated according to the following expression:


$$Re_{p}=\rho_{f}|u-v_{p}|d_{p}/\mu_{f}$$ (9)

$$C_{D}=\psi (\frac{200\alpha_{s}}{\alpha_{f}\phi^{2}Re}+\frac{7}{3\phi })+(1-\psi )\alpha_{f}^{-1.65}max\{\frac{24}{\alpha_{f}Re_{p}}[1+0.15{\left(\alpha_{f}Re_{p}\right)}^{0.687}],0.44\}$$ (10)

where $Re_{p}$ is defined as the particle relative Reynolds number, which depends on the particle diameter, fluid viscosity, and particle sphericity. Re represents the Reynolds number. The mixing parameter *ψ*, defined as a function of the fluid volume fraction $\alpha_{f}$, and $\alpha_{s}$ represents the local volume fraction of the solid phase at the current time step.

### 2.3. Coupling method

**Fig 1:**
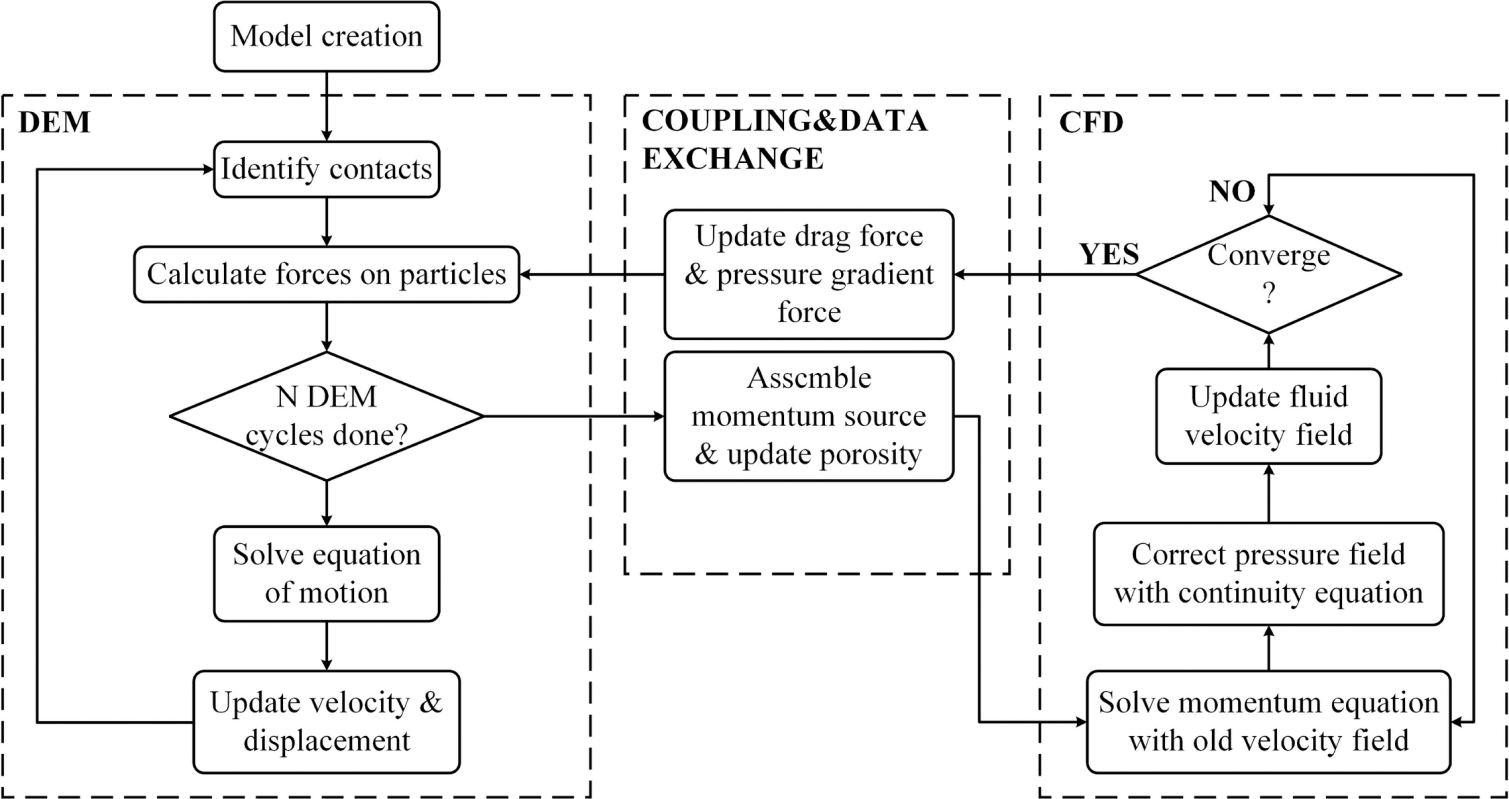
CFD-DEM Coupling Scheme (Modified from Qian [30]).

The coupling procedure between the particles and the fluid is implemented using an interleaved scheme, enabling parallel calculations of both the DEM and CFD. In the DEM, the particle positions are updated to calculate the solid volume fraction for the fluid cells. The CFD then iteratively solves for the flow velocity and pressure within each cell to calculate the solid-fluid interaction forces, based on the volume fraction obtained through the Euler-Lagrange mapping. Subsequently, the DEM updates the particle positions again for the next cycle, according to the forces acting on particles, including both inter-particle contact forces and particle-fluid interaction forces. The coupled computational process is illustrated in Fig 1.

### 2.4. Validation

**Fig 2:**
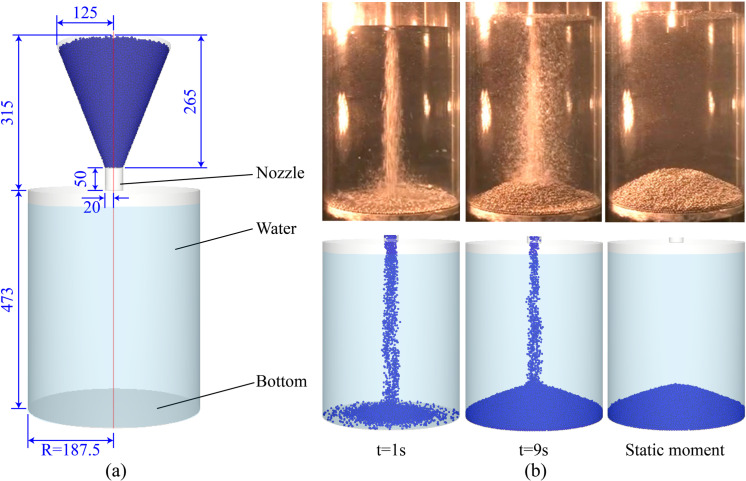
Model parameters and simulation results. (a) Three-dimensional schematic diagram of the model (unit: mm); (b) Comparison of particle settling during the experiment (top: Sheikh et al.‘s experiment [48], bottom: CFD-DEM numerical simulation experiment).

To validate the feasibility and accuracy of the CFD-DEM coupling method and the drag model in simulating solid-solid collisions and fluid-solid interactions, this section presents numerical simulations of underwater granular deposition experiments and compares the results with the experimental data from Sheikh et al. [48]. The setup, shown in Fig 2a, consists of a cylindrical water storage tank with a diameter of 375 mm and a granular injection funnel equipped with a 40 mm diameter nozzle at the bottom. At the beginning of the numerical simulation, the nozzle is opened, allowing particles to sink freely under gravity into the water, thereby forming a slope at the bottom of the tank. The DEM parameters are set based on the experimental conditions described by Sheikh et al., as shown in Table 1.

**Table 1:** DEM numerical simulation parameters (adapted from Sheikh [48]).

Parameters	Values
Density of particles (kg/m^3^)	7800
Young’s modulus of particles (Pa)	5.4 × 10^6^
Poisson’s ratio of particles	0.305
Coefficient of restitution (solid–solid collision/solid–wall collision)	0.30
Coefficient of static friction (solid–solid collision/solid–wall collision)	0.25
Drag Law	Huilin & Gidaspow
Time step of particles	1.78 × 10^-5^

**Fig 3:**
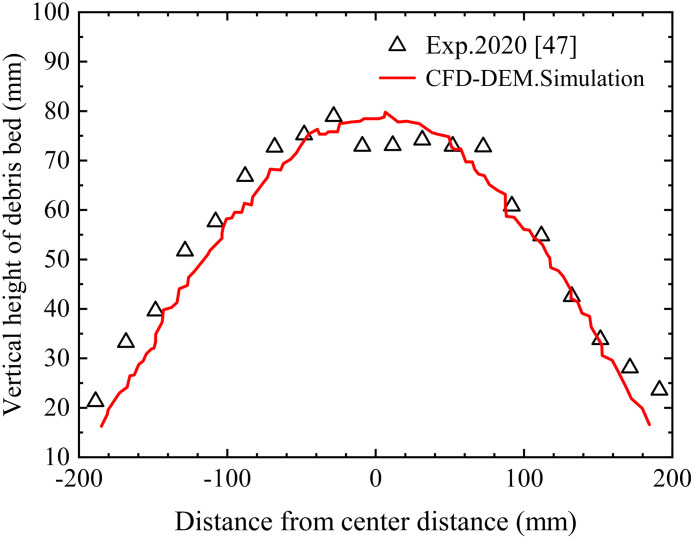
Profile height comparison of the accumulated slope.

Fig 2b shows snapshots of CFD-DEM simulations and physical experiments at different time steps. It can be observed that the particle distribution and slope shape are highly similar between the two. Fig 3 presents a comparison of the profile heights of the accumulated slopes, demonstrating that the slope shape formed in the CFD-DEM simulation closely matches that of the physical model experiment. This comparison clearly indicates that the CFD-DEM coupling method and the drag model employed in this study are effective in replicating solid-solid collisions and fluid-solid interactions. The solid-fluid coupling method and drag model will be further extended to simulate complex progressive failures of tunnel face as described below.

**Fig 4:**
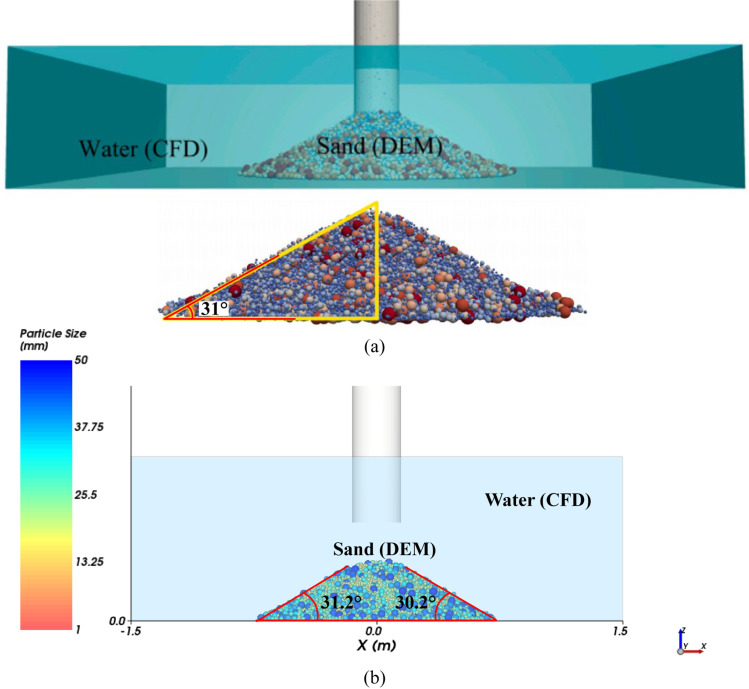
Comparison of CFD-DEM modeling the collapse of underwater sand column. (a) CFD-DEM simulation by Fu et al. (2022). (b) CFD-DEM simulation in this study.

To further validate the CFD-DEM method employed in this study, the method has been also used to reproduce a previous CFD-DEM numerical case of the collapse of underwater sand column reported by Fu et al. [29] based the same model parameters, as shown in Fig 4. The natural repose angle of 31.2° has been found as presented in Fig 4b, close to 31° reported by Fu et al. as given in Fig 4a.

## 3. Simulation procedure

### 3.1. Material parameters

**Fig 5:**
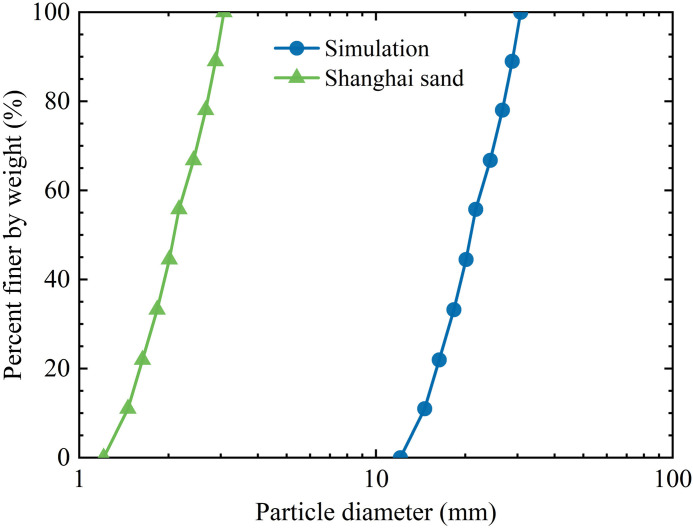
The Shanghai sand particle size distribution and the particle size distribution adopted in the simulation.

Spherical particles are used in the DEM simulation. The particle size distribution refers to that used by Yin et al. [49]. For actual engineering scale, the number of particles may reach tens of millions, which can exceed the current DEM computing capabilities. To address this, the particle size is increased by a factor of ten to reduce the number of particles, a common approach in DEM simulations [21,26,50]. The largest and smallest particle sizes in the simulation are 30.7 mm and 12.2 mm, respectively. The particle size distributions for both Shanghai sand and the simulation model are shown in Fig 5. The contact model for the DEM part is the Mindlin-Hertz model. A particle density of 2650 kg/m³ and a friction coefficient of 0.5 are adopted, as suggested by previous studies [30,51]. Additionally, the Young’s modulus in the contact model is set to 5 × 10^8^ N/m^2^ based on trial calculations, ensuring that the particle overlap does not exceed 2% of the total particle volume throughout the whole simulation [24,26,28]. In the CFD simulation, the computational domain must be large enough to avoid boundary effects from the sidewalls, but also small enough to reduce computational costs. Based on prior research, the CFD computational domain is designed to fully encompass the specimen, with a width-to-particle *d*_50_ ratio of 30 to mitigate sidewall effects [52].The fluid mesh size is set to 100 mm, approximately five times the *d*_50_ value, in order to balance both convergence and resolution of the fluid calculations [53]. The material properties of the fluid phase are based on pure water at 100 kPa and standard temperature. Following Fu et al. [29], the fluid density is set to 998.2 kg/m³, and the viscosity is 0.001003 Pa·s. To ensure simulation efficiency while maintaining accuracy and stability, the DEM time step is set to 2 × 10^−7^ s, while the CFD time step is set to 2 × 10^−5^ s, corresponding to 100 times that of the DEM [30]. Additional simulation parameters are provided in Table 2.

**Table 2:** Input parameters for CFD-DEM simulation.

Parameters	Values	Unit
DEM		
Density of particles	2650	kg/m^3^
Young’s modulus of particles	5 × 10^8^	Pa
Poisson’s ratio of particles	0.3	
Inter-particle friction coefficient	0.5	
Rolling friction coefficient	0.1	
Number	470000	
Restitution coefficient	0.3	
Drag Law	Huilin & Gidaspow	
Timestep	2 × 10^−7^	s
CFD		
Model	Laminar flow	
Fluid density	998.2	kg/m^3^
Fluid viscosity	0.001003	Pa·s
Gravity of water	98.1	m/s^2^
Mesh size	100	mm
Timestep	2 × 10^−5^	s

### 3.2. Simulation process

In this paper, the coupled CFD-DEM method is used to simulate the progressive failure of the tunnel face in saturated soil. Four tests are performed using the same model size, with the tunnel surrounded by both dry and saturated sandy soils. The failure occurs in the form of both forward and backward tunnel movement.

**Fig 6:**
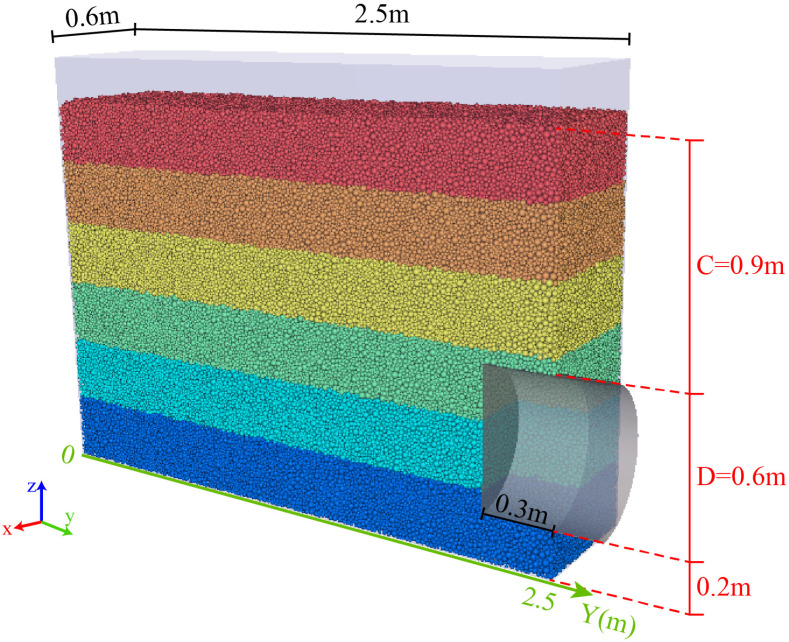
The CFD-DEM model to simulate the progressive failure of tunnel face (C represents the depth of the tunnel and D represents the diameter).

Fig 6 shows the schematic diagram of the discrete element part of the coupled model. Due to the symmetry of the simulation setup, half of the tunnel model is used to save computational resources [49]. To enhance computational efficiency, following the studies of Jiang [54] and Yin [49], all particles in this study are compacted under a simulated gravity of 10g, thus reducing the simulation model size to one-tenth of the actual prototype and decreasing the number of particles required for the calculation. The friction coefficient is set to 0.5 for both particle-to-wall and particle-to-particle interactions. In an ideal case, particles on both sides of the symmetry plane exhibit identical translational velocities, with no relative motion or friction. The symmetry plane is assumed to be smooth, and the friction coefficient between the tunnel face and the particles is also set to 0.

In this study, the numerical simulations consist of the following four stages:

(1) Specimen preparation: Specimens are generated using the layered under-pressure method [54,55] to ensure initial uniformity, with an acceleration set to 98.1 m/s^2^, which is equivalent to 10 times the acceleration due to gravity.
(2) Tunnel penetration: Insert the tunnel wall into the specimen and remove the particles within the excavation area. Subsequently, recalculate until the system reaches equilibrium.
(3) Coupling fluid dynamics: For the saturated sandy soil specimen, the CFD computational domain is introduced to perform coupling calculations, followed by the recalculation of equilibrium.
(4) Tunnel face movement: The translational velocity of the tunnel face is applied to simulate the progressive failure process of the tunnel excavation surface. For the saturated sandy soil, this step involves coupling the CFD model to perform parallel calculations.

Due to the complexity of controlling excavation speed in practical projects, this paper simplifies the active and passive failure modes into two cases: tunnel movement in the backward and forward directions. Consistent with previous numerical simulations and experiments, the tunnel excavation speed is set to a constant value of 0.01 m/s for both forward and backward movements to satisfy the quasi-static conditions. The total simulation time is 20 s, and the distance covered in both the forward and backward movements is 200 mm.

**Fig 7:**
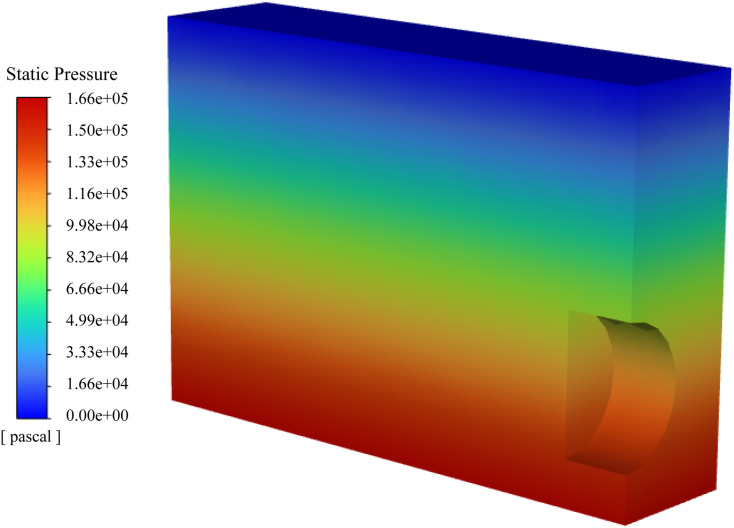
Initial water pressure distribution in the CFD model.

**Fig 8:**
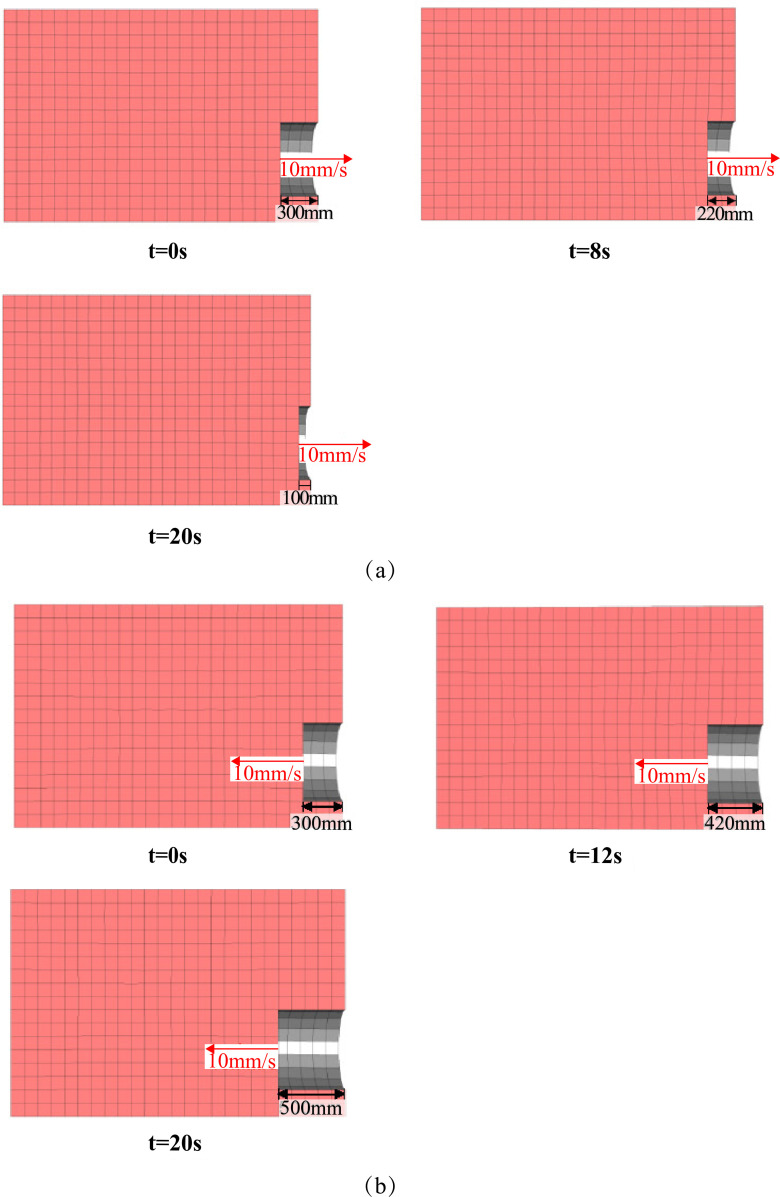
Schematic diagram of CFD dynamic mesh for two damage modes. (a) CFD mesh change for backward tunnel movement. (b) CFD mesh change for forward tunnel movement.

In the CFD model, the tunnel is set as a wall boundary condition. The tunnel excavation surface is set as a velocity boundary with a constant speed of 0.01 m/s, which corresponds to the excavation speed used in the DEM simulation. The top boundary is set as a 0-velocity inlet, simulating the static groundwater level at the ground surface. The remaining boundaries are set as pressure boundary conditions, with pressure values determined by the local hydrostatic head. The initial water pressure distribution is shown in Fig 7. During the progressive failure of the tunnel face, the groundwater coverage changes in parallel with the movement of the tunnel. To simulate this phenomenon, a dynamic mesh approach is employed in the CFD section. The boundary of the tunnel face in the CFD simulation moves synchronously with the excavation progress in the DEM simulation (as shown in Fig 8), ensuring the accuracy of the coupled simulation.

## 4. Results analysis

### 4.1. Progressive failure

**Fig 9:**
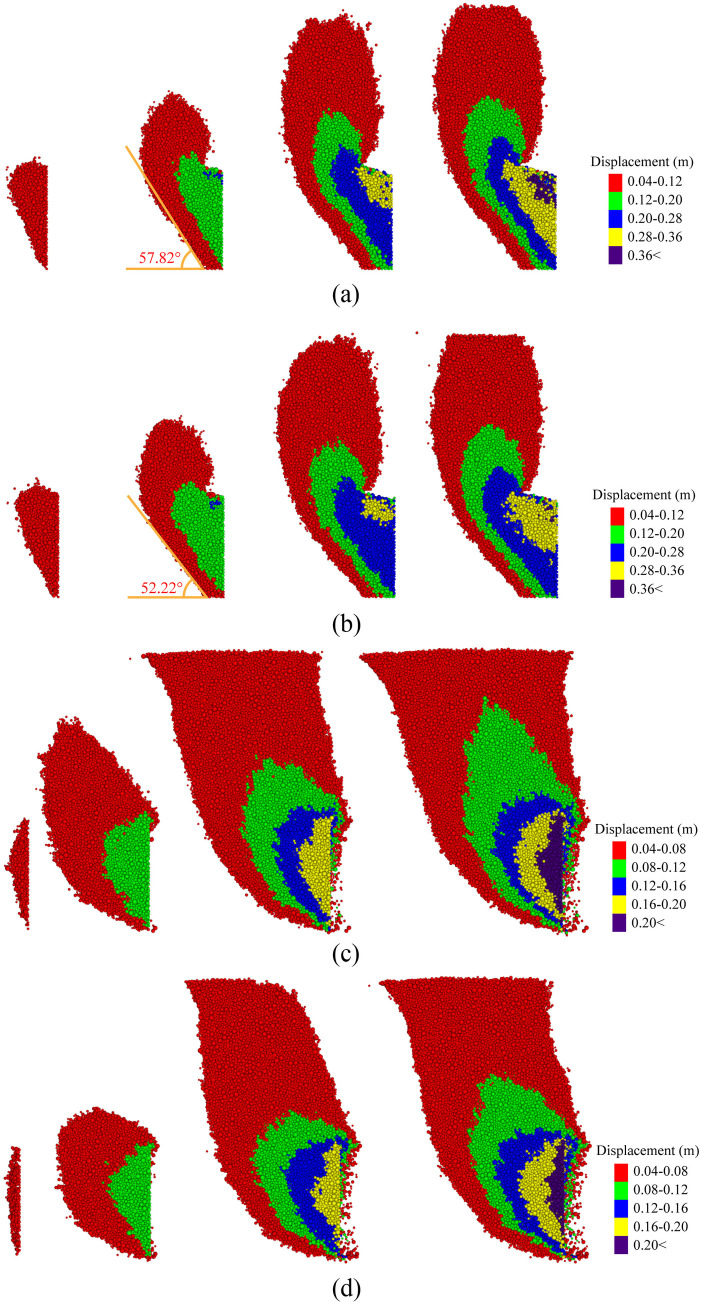
Particle displacement fields from left to right for 40mm, 100mm, 170mm, and 200mm tunnel displacements. (a) Backward displacement in saturated sand. (b) Backward displacement in dry sand. (c) Forward displacement in saturated sand (d) Forward displacement in dry sand.

Fig 9 shows the distribution of particle displacement in four tests with different tunnel excavation distances (20 mm, 100 mm, 170 mm, 200 mm). Particles with a displacement of less than 0.04 m are omitted for clarity. Firstly, in the active failure mode of the tunnel face, it can be observed from Fig 9a and 9b that as the tunnel face moves, the destabilization zone gradually expands, and the displacement of particles in this zone increases. Gradually, a ‘wedge + prism’ destabilization zone forms at the front of the tunnel face. For the case with a cover-to-diameter ratio (C/D) of 1.5, the destabilization zone does not fully extend to the ground by the end of the test, which is consistent with the results of previous centrifuge tests [56,57].

When comparing the displacement fields formed by tunnel face movement in dry sand (Fig 9a) and saturated sand (Fig 9b), the shapes are similar; however, the destabilization zone in dry sand is more extensive than in saturated sand. The destabilization angle in saturated sand (57.82 °) is slightly larger than that in dry sand (52.22 °), which is consistent with the results of previous tests [58].

As depicted in Fig 9c and 9d, in contrast to the backward movement of the tunnel face, the destabilization zone extends to the ground surface in both dry sand and saturated sand. When comparing the displacement fields in these two soil conditions, the size of the instability zone shows little difference at the initial stages of the tunnel face movement (40 mm, 100 mm). However, as the test progresses, a significant difference in the size of the displacement field develops between the two conditions. Specifically, it is evident that the destabilization zone in the saturated sand is significantly larger than that in the dry sand.

**Fig 10:**
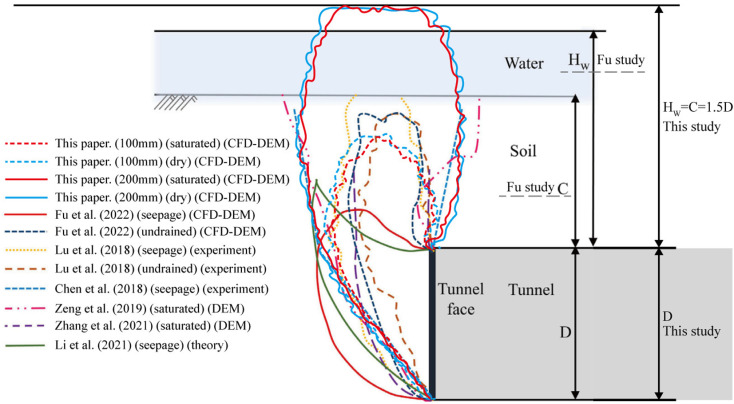
Comparison of failure mechanisms with other solutions. (Modified from Fu et al. [29]).

Fig 10 shows the relevant experiments done by predecessors. Considering that the cover depth in our study is greater than that of Fu et al., we have compared the displacement field for tunnel retreats of 100 mm and 200 mm with the results of Fu et al. [29], and conducted a qualitative analysis. As shown in Fig 10, during the mid-simulation stage (with a tunnel retreat of 100 mm), the soil instability zone exhibits a closed morphology similar to that observed by Zhang et al. [20]and Fu et al. [29], without reaching the ground surface. As the simulation progresses (with a tunnel retreat of 200 mm), the instability zone gradually expands to the surface, displaying a funnel shape similar to the one observed by Zeng et al. [21] and Chen et al. [59]. The critical soil instability zone ahead of the tunnel is consistent with the observations of Zeng et al. [21] and Chen et al. [59]. The entire process illustrates the development of the soil instability region. Moreover, the instability zone in dry sand is slightly larger than in saturated sandy soil, which is in agreement with the findings of Zeng et al. [21].

**Fig 11:**
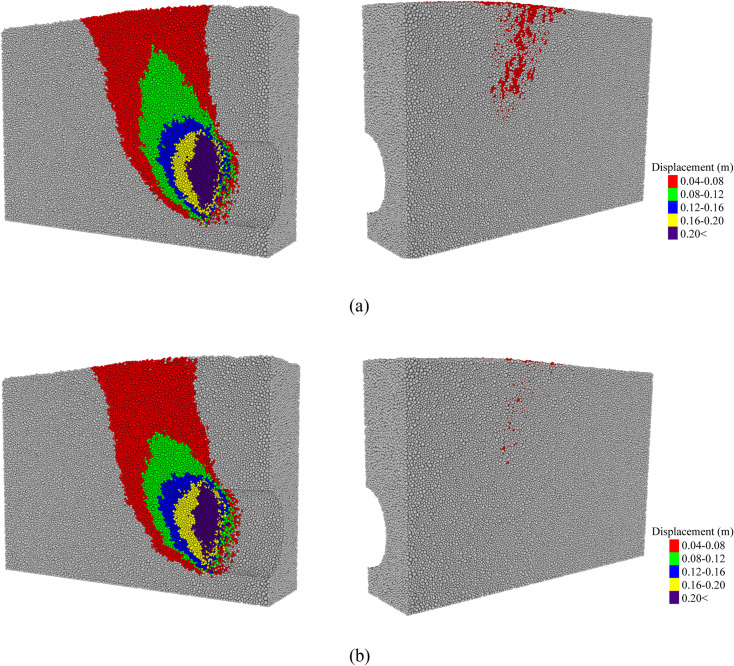
Complete particle displacement field at 200 mm of tunnel face displacement. (a) In saturated sand. (b) In dry sand.

Fig 11 shows the displacement fields of the DEM model for all particles. As shown in Fig 11a and 11b, the destabilization zone in the saturated sand extends to the back of the model, while the destabilization zone in the dry sand remains unobservable. This observation further suggests that the destabilized area in the saturated sand is significantly larger than that in the dry sand in the passive failure mode. It also indicates that the development of the soil displacement field in the passively damaged saturated sand model is influenced by the boundary.

### 4.2. Ground surface displacement

**Fig 12:**
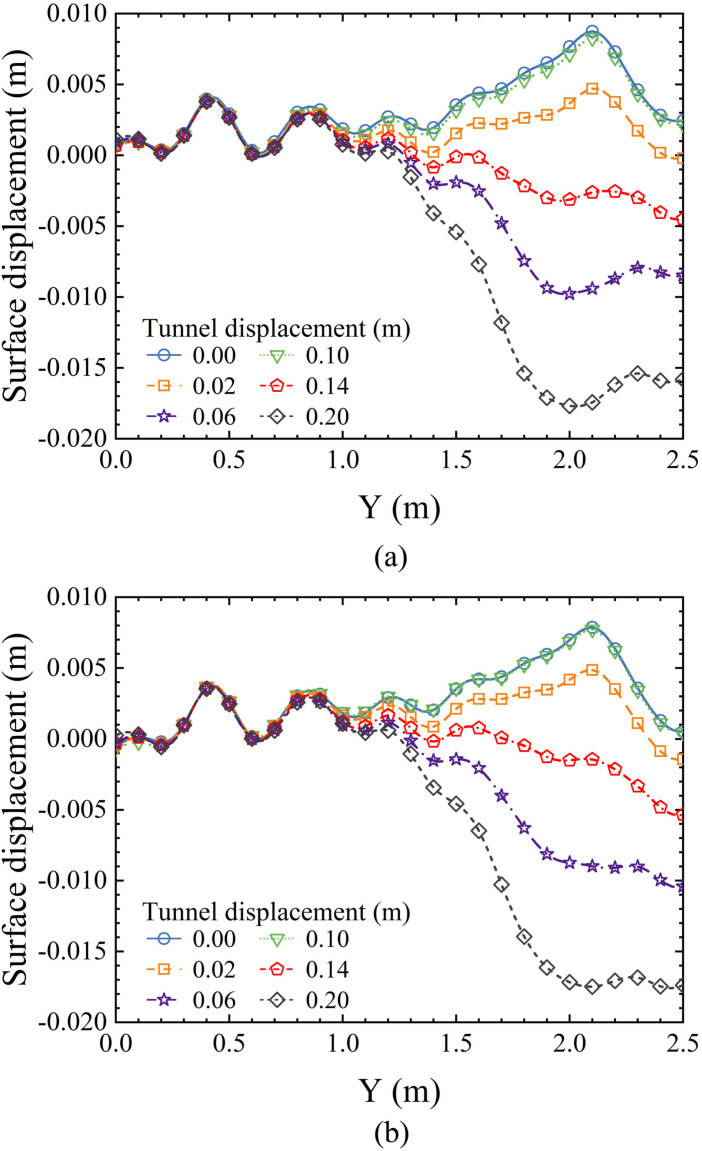
The surface settlement caused by tunnel face backward movement. (a) In dry sand. (b) In saturated sandy soil.

Surface displacements caused by tunnel excavation may affect nearby buildings, which is a major concern in urban tunnel construction. The surface displacements resulting from tunnel face failure are often more significant than anticipated and require particular attention. Fig 12 presents the surface displacements for different tunnel face backward movements under two soil conditions: saturated sand and dry sand. It is observed that surface settlement increases with tunnel movement, reaching its maximum value by the end of the test.

The maximum settlement in dry sand is observed at Y = 2.025 m, with settlement decreasing rapidly on both sides of the maximum, forming a V-shaped curve on the graph. In saturated sand, the maximum settlement occurs at Y =  2.1 m, which is similar to that in dry sand. In contrast, the settlement on either side of the maximum in saturated sand decreases more gradually, resulting in a greater overall settlement than in dry sand.

**Fig 13:**
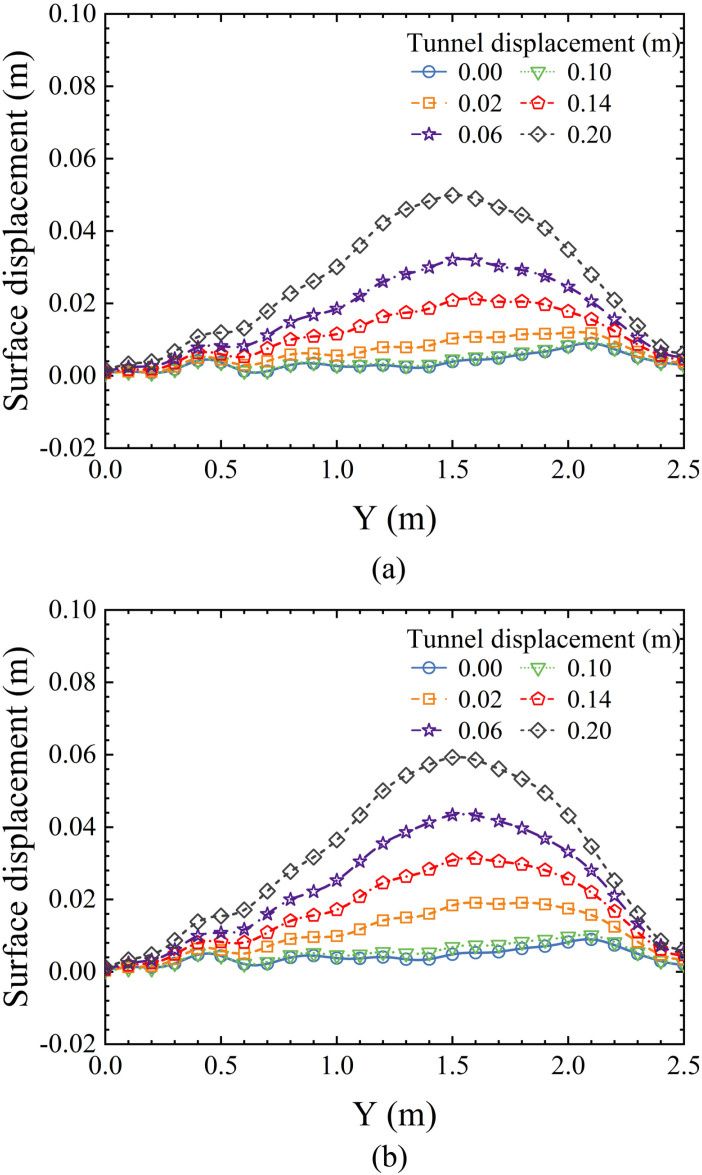
The surface uplift caused by tunnel face forward movement. (a) In dry sand. (b) In saturated sandy soil.

Fig 13 shows the surface uplift corresponding to two soil conditions—saturated sand and dry sand—during the forward movement of the tunnel. In both soil conditions, the surface uplift increases as the tunnel advances. The uplift is minimal at both ends and peaks in the middle, with the maximum uplift occurring at Y = 1.5 m in the model. Additionally, the surface uplift in dry sand is smaller than that in saturated sand. This is consistent with previous results on the displacement field [58,60,61], which suggest that passive failure in saturated sandy soil conditions leads to a greater extent of soil movement and deformation, resulting in larger hazards.

### 4.3. Supporting force-displacement curve

**Fig 14:**
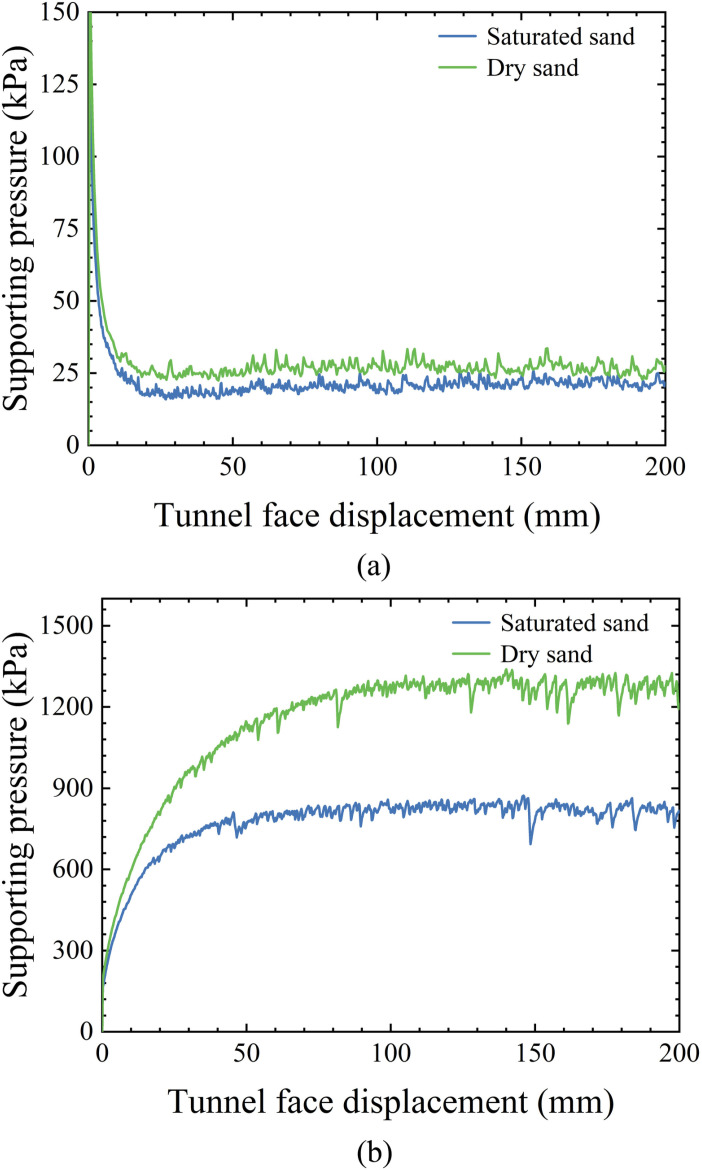
The curve of the tunnel face support force versus tunnel displacement. (a) In dry sand. (b) In saturated sandy soil.

The curve of the tunnel face support force versus tunnel displacement is shown in Fig 14, indicating that under the active damage case, the curve has two stages: in the first stage, the support force decreases rapidly as the tunnel face displacement increases, and in the second stage, the support force stabilizes and reaches the ultimate value. Comparing the two curves, the ultimate support force in both saturated sand and dry sand is relatively small, due to the formation of an arch in the soil. More details about the force chain and arch formation will be analyzed in the next section. In contrast, for the passive failure curve, the support force is much greater than that in the active damage case. Unlike the active damage mode, the support force does not experience a sharp decrease but instead increases gradually to a limiting value as the tunnel face displacement increases.

### 4.4. Force chain and stress distribution

By utilizing the properties of DEM, micromechanical properties can be captured at the particle scale. For example, the force chains formed by particle contacts can be visualized, providing crucial microscopic characterization for multiscale models. During progressive tunnel failure, this visualization allows for a more intuitive observation and analysis of soil arch formation characteristics.

**Fig 15:**
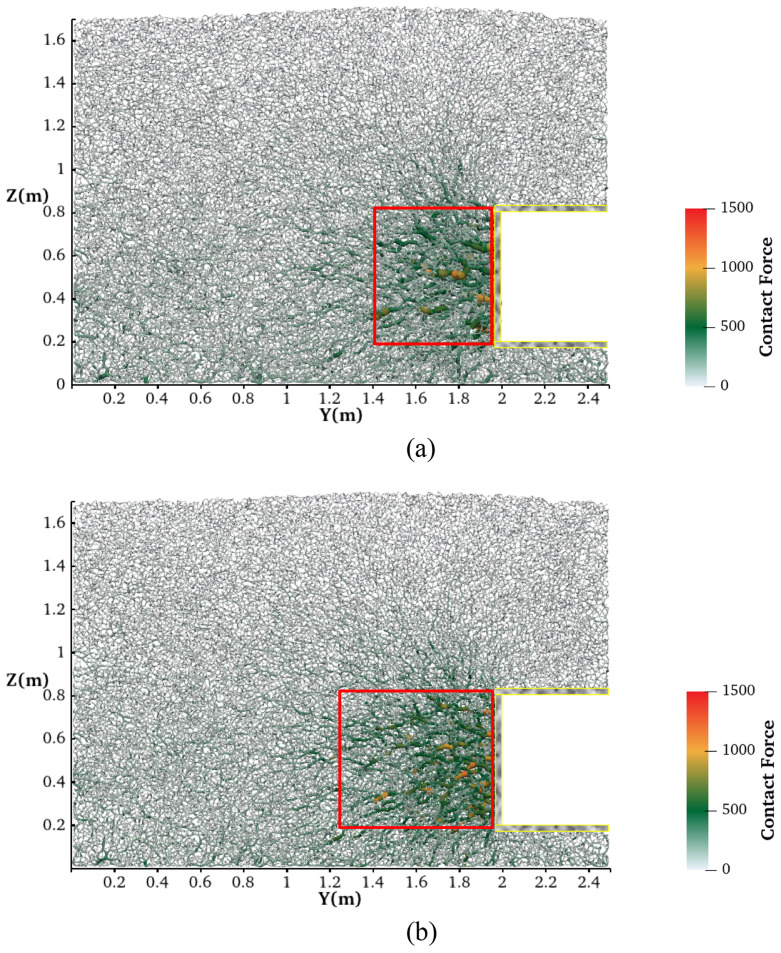
Force chain distribution after tunnel face forward movement of 200mm. (a) In saturated sand. (b) In dry sand.

Fig 15 shows the force chain diagram for the passive failure tests at the tunnel face forward displacement of 200 mm. The starting and ending positions of each force chain in the figure are the spherical center of the contact particles. The force chains’ corresponding magnitudes are indicated by the color, transparency, and thickness. During the forward movement of the tunnel, the surrounding soil undergoes compression, resulting in a significant increase in lateral contact forces. As illustrated within the red box in the figure, there is a notable increase in both the number and magnitude of the force chains near the tunnel face, accompanied by a shift in their orientation towards the horizontal direction. A comparison of the force chain development between saturated sand and dry sand reveals a significantly larger horizontal distribution area of strong force chains (denoted by the red box) in dry sand. This can be attributed to the fact that, in saturated sand, a portion of the applied pressure is carried by the pore water between the particles, whereas in dry sand, the soil particles themselves are responsible for supporting the entire pressure at the tunnel face. Additionally, the strong force chains in the dry sand are mainly distributed at the tunnel's front end.

**Fig 16:**
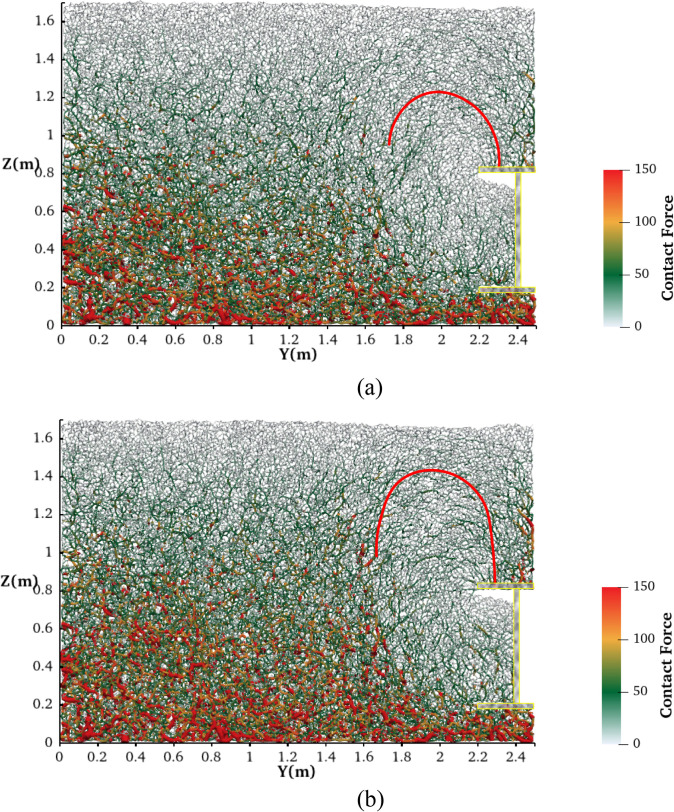
Force chain distribution after tunnel face backward movement of 200mm. (a) In saturated sand. (b) In dry sand.

Fig 16 shows the force chain diagram for the active failure tests at the tunnel face backward displacement of 200 mm. Although previous studies have shown that soil forms an arch above the excavation face during active failure, the conclusion in the indoor test is mainly identified by the displacement field image. In contrast, the soil arch area can be more clearly observed through the force distribution visualized by DEM [21,54,58]. As depicted by the red curves in the figure, soil arches are present under both soil conditions. However, the contours of soil arches in dry sand are more pronounced, and their positions are slightly higher compared to those in saturated sand. This observation suggests that stronger and higher soil arches are more easily formed in dry sand. Once a significant amount of soil has entered the tunnel, the presence of these soil arches enables soil particles located far from the excavation face to maintain equilibrium, thereby mitigating surface subsidence. Consequently, under conditions where the working face retreats, the surface subsidence in dry sand is slightly less than that in saturated sand, which is consistent with the displacement field analysis above.

**Fig 17:**
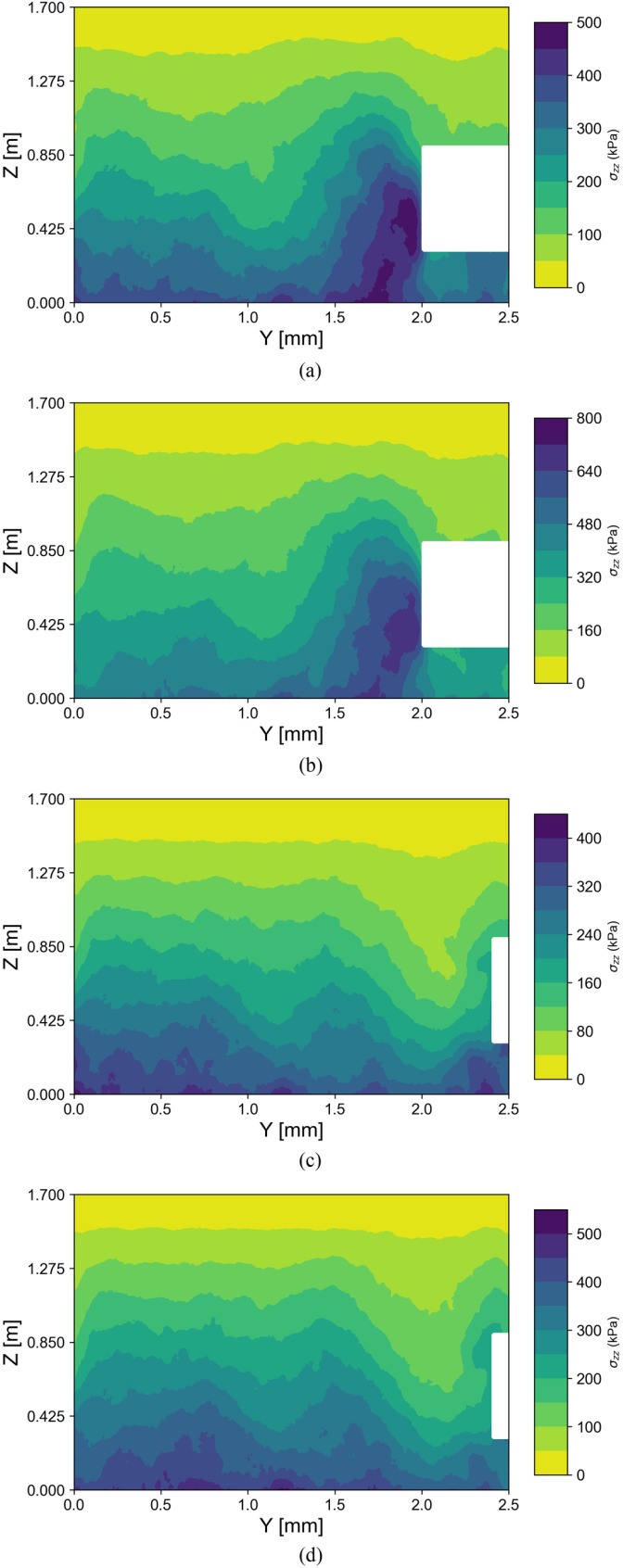
Stress distribution in the model. (a) Passive failure in saturated sand. (b) Passive failure in dry sand. (c) Active failure in saturated sand. (d) Active failure in dry sand.

Fig 17 shows the distribution of vertical stress when the tunnel face is displaced by 200 mm. It can be observed that, in the passive failure mode, the vertical stress distribution pattern is similar for both soil conditions, with the front side of the tunnel face causing a significant increase in vertical stress. When one moves away from the tunnel face, the vertical stresses are less affected by the tunnel face's instability and tend to increase with depth. However, the maximum vertical stress in dry sand is significantly higher than that in saturated sand. In the active failure mode, the vertical stress decreases, but it remains higher in dry sand compared to saturated sand. An abrupt reduction in stress at the left-end boundary is observed in all four tests, indicating that the horizontal model length should be increased to eliminate this boundary effect.

## 5. Conclusion

In this paper, the coupled CFD-DEM method and the dynamic mesh method are used to simulate the progressive failure of the tunnel face under two soil conditions: saturated sand and dry sand. Two failure modes are simulated: active failure and passive failure. At the macroscopic level, the displacement field of soil particles, surface displacement variations, and the development of support forces are presented throughout the failure process. The differences in these macroscopic phenomena between the two soil conditions and the two failure modes are compared. Additionally, the changes in force chains and stress distribution are analyzed to reveal the underlying microscopic mechanisms. The main conclusions are summarized as follows:

(1) In the active damage mode, a ‘wedge + prism’ soil distribution characteristic is present at the front end of the excavation. The destabilization zone does not completely extend to the ground surface in either of the two soil conditions. The destabilization angle in saturated sand is slightly larger than in dry sand. However, in the passive failure mode, the destabilization zone extends completely to the surface and is significantly larger in saturated sand compared to dry sand.
(2) In the active failure mode, the maximum surface settlement value in saturated sand is similar to that in dry sand, and the overall settlement in saturated sand is greater than that in dry sand. In the passive failure mode, both the maximum surface uplift and the uplift range in the saturated sand are larger than those in the dry sand.
(3) In the active failure mode, the ultimate support force at the excavation face is similar in saturated sand and in dry sand. However, in the passive failure mode, the ultimate support force at the excavation face is higher in dry sand than in saturated sand.
(4) In the active failure mode, a soil arch forms above the excavation face, which is more pronounced in dry sand than in saturated sand. In the passive failure mode, a strong chain zone is forms near the tunnel face to withstand significant horizontal support forces, which is stronger in dry sand than in saturated sand.
